# Long-Term Liver-Targeted AAV8 Gene Therapy for Mucopolysaccharidosis IVA

**DOI:** 10.3390/cimb47110900

**Published:** 2025-10-29

**Authors:** Shaukat A. Khan, Eliana Benincore-Florez, FNU Nidhi, Jose Victor Álvarez, Dione A. Holder, Shunji Tomatsu

**Affiliations:** 1 Department of Biomedical Research, Nemours Children’s Health, Wilmington, DE 19803, USA; shaukat.khan@nemours.org (S.A.K.); elianabenincore@javeriana.edu.co (E.B.-F.); nnidhi@udel.edu (F.N.); jose.victor.alvarez.gonzalez@sergas.es (J.V.Á.); dione.holder@nemours.org (D.A.H.); 2 Institute for the Study of Inborn Errors of Metabolism, Faculty of Science, Pontificia Universidad Javeriana, Bogotá 110231, Colombia; 3 Department of Biological Sciences, University of Delaware, Newark, DE 19716, USA; 4 Department of Pediatrics, Hospital Clínico Universitario de Santiago de Compostela, Health Research Institute of Santiago de Compostela (IDIS), CIBERER, MetabERN, 15706 Santiago de Compostela, Spain; 5 Department of Pediatrics, Shimane University, Izumo 693-8501, Japan; 6 Department of Pediatrics, Graduate School of Medicine, Gifu University, Gifu 501-1193, Japan; 7 Department of Pediatrics, Thomas Jefferson University, Philadelphia, PA 19107, USA

**Keywords:** AAV, gene therapy, MPS IVA, GALNS, skeletal dysplasia

## Abstract

Mucopolysaccharidosis IVA (MPS IVA) is a lysosomal storage disease with an autosomal recessive trait caused by the deficiency of N-acetylgalactosamine-6-sulfate sulfatase (GALNS) enzyme, which leads to the accumulation of chondroitin-6-sulfate and keratan sulfate, primarily in cartilage and its extracellular matrix, resulting in a direct impact on cartilage and bone development, as well as subsequent systemic skeletal dysplasia. ERT and HSCT are current treatment options, but they have a limited effect on bone lesions. In this article, we investigated liver-specific AAV8 vectors with a thyroxine-binding globulin promoter in the MPS IVA murine model to evaluate the long-term (24 weeks in males and 48 weeks in females) effects of gene therapy on biochemical markers and bone pathology. Both treated groups showed GALNS enzyme activity at supraphysiological levels in plasma and in various tissues, including the liver, heart, spleen, and bone. Keratan sulfate in both groups was normalized in plasma, liver, and bone (male mice). Pathological analyses revealed a decrease in vacuolated cells in the heart muscle and valves and improvement in bone pathology in treated male mice. However, the therapeutic impact was less pronounced in treated female mice. Overall, male mice indicated a substantial improvement in biochemical parameters and pathology compared to female mice.

## 1. Introduction

Mucopolysaccharidosis type IVA (MPS IVA or Morquio A syndrome) is an autosomal recessive lysosomal disorder caused by mutations in the N-acetyl-galactosamine-6-sulfate sulfatase (GALNS) gene, leading to a deficiency of the GALNS enzyme [1,2,3,4,5]. This defect causes glycosaminoglycans (GAGs), such as chondroitin 6-sulfate (C6S) and keratan sulfate (KS), to accumulate in tissues like cartilage, bone, connective tissues, the heart, and the cornea, resulting in severe skeletal dysplasia [6,7,8,9]. Although infants with MPS IVA appear normal at birth, progressive skeletal issues usually develop around age 3. Clinical signs include short stature with a short neck and trunk, odontoid hypoplasia causing neck instability, pectus carinatum, tracheal narrowing, obstructive and restrictive lung diseases, kyphoscoliosis, joint laxity, coxa valga, genu valgum, and gait problems [10,11,12]. Without treatment, many patients become disabled and require wheelchairs during their teens. Skeletal deformities, including imbalance of growth, can block the trachea, leading to high mortality and morbidity; about two-thirds die from respiratory failure, and one-third from heart problems [9,13,14,15]. Current options include enzyme replacement therapy (ERT) with elosulfase alfa [10,16,17] and hematopoietic stem cell transplantation (HSCT) [10,18,19]. While these improve daily function, they rarely affect skeletal abnormalities. ERT has limitations, such as weekly infusions lasting 4–6 h, rapid clearance with a short half-life (35 min in humans, 2 min in mice) [20,21], and high costs [22,23]. Additionally, patients on ERT show no remission of bone problems [14,22,24,25,26], which is consistent with findings in ERT-treated MPS IVA mice [21,27]. HSCT has its own constraints, including the need for a matched donor, age limits, limited specialized personnel, and risks such as graft-versus-host disease (GVHD), infections, and other complications [18,19,24,28,29]. Although HSCT may enhance quality of life, its effect on bone lesions remains unclear [30]. Alternative therapies include pharmacological chaperones [31], substrate reduction therapy [32], nanoparticles [10], CRISPR/Cas9 gene editing [33], ex vivo lentiviral gene therapy [34], and AAV-based gene therapy [35,36]. AAV vectors contain two viral genes, rep (for replication) and cap (for capsid), flanked by inverted terminal repeats (ITRs). Different AAV serotypes target tissues differently due to variations in the cap open reading frame (ORF), which can cause different immune responses and transduction efficiencies [37,38,39]. Some serotypes are better suited for specific organs; for example, AAV8 is highly efficient in liver cells, demonstrating superior transduction of hepatocytes in mice, dogs, and non-human primates (NHPs) [40,41,42,43,44,45,46,47,48]. Systemically delivered in mice, AAV8 effectively transduces skeletal and cardiac muscle [49]. AAV gene therapies are promising due to their safety, efficiency, and the potential for sustained transgene expression [50,51]. Rossi et al. showed that AAV gene therapy is safe and effective for mucopolysaccharidosis VI, suggesting potential for other mucopolysaccharidoses [50]. Currently, several AAV-mediated gene therapies for MPS I, II, IIIA, IIIB, and VI are in clinical trials (clinicaltrial.gov).

We previously reported that in models of MPS IVA, intravenous administration of an AAV8 vector encoding a human GALNS (hGALNS) under a liver-specific promoter (thyroxine-binding globulin [TBG]) resulted in supraphysiological enzyme levels in plasma and partial improvement of bone pathology [35]. Recently, we described the use of AAV8 or AAV9 vectors with tissue-specific and ubiquitous promoters in MPS IVA mice to compare therapeutic efficacy based on biochemical markers and bone pathology [36]. All vectors studied showed significant improvements in biochemical parameters and pathology. Previously, we conducted a 12-week follow-up study after AAV-mediated gene therapy (at 16 weeks old) to assess therapeutic efficacy [35,36], which revealed supraphysiological enzyme levels in plasma and partial correction of bone and cardiovascular issues. Our goal was to determine whether AAV8-mediated gene therapy, beyond 16 weeks, maintains GALNS expression, reduces pathological storage, and improves bone and cardiovascular conditions. Therefore, in the current study, we evaluated long-term therapy using an AAV8 vector (REGENXBIO, Rockville, MD, USA) with a liver-specific promoter (thyroxine-binding globulin [TBG]) in the MPS IVA mouse model. We designed two groups of mice for AAV8-TBG treatment, choosing 24 weeks for male mice and 48 weeks for female mice. Untreated male and female mice served as controls in each respective group.

## 2. Materials and Methods

### 2.1. Cassette Design and Expression of AAV Vector Production

The AAV8-TBG-hGALNS vector was produced using a scaled-down version of the proprietary GMP vector manufacturing protocols (REGENXBIO, Rockville, MD, USA). Briefly, HEK293 cells (RGX293) were triple-transfected with the helper plasmid, the AAV8 capsid plasmid, and the transgene plasmid containing hGALNS. The packaged vectors were purified from the cell culture supernatant through affinity chromatography and quantified using the Droplet Digital PCR (Bio-Rad, Hercules, CA, USA) method.

### 2.2. Animal Experimentation

We used GALNS knockout (KO, Galns^−/−^) MPS IVA mouse models on the C57BL/6 background. KO mice showed no detectable GALNS enzymatic activity in blood and tissues and exhibited excessive accumulation of storage materials, mainly within reticuloendothelial Kupffer cells, muscle, heart valves, and chondrocytes, including articular cartilage and the growth plate [52].

Homozygous male and female MPS IVA mice at 4 weeks old were treated with AAV vectors at a dose of 5 × 10^13^ GC/kg via the lateral tail veins using the TBG promoter (AAV8-TBG-hGALNS). The male mice were 24 weeks old and the female mice were 48 weeks old at the endpoints. The second group of MPS IVA KO mice received phosphate-buffered saline (PBS). This group also included male (24 weeks) and female mice (48 weeks). Wild-type (WT) male (24 weeks) and WT female (48 weeks) mice were administered PBS. The total volume administered was approximately 100 μL per mouse. Around 100 μL of blood was collected in EDTA tubes (Becton Dickinson, Franklin Lakes, NJ, USA) every other week from all animals. The blood was centrifuged at 8000 rpm for 10 min, and the separated plasma was stored at −20 °C until the GALNS enzyme and GAG assays were performed. At 24 weeks for males and 48 weeks for females, mice were euthanized in a CO_2_ chamber and perfused with 20 mL of 0.9% saline. Liver, heart, lung, muscle, trachea, spleen, kidney, and knee joint were collected and stored at −80 °C until analysis for GALNS enzyme activity and GAG content. Knees were also stored in 10% neutral buffered formalin for histopathology. All animal care and procedures adhered to NIH guidelines and received approval from the Institutional Animal Care and Use Committee of Nemours Children’s Health.

### 2.3. GALNS Enzyme Activity Assay

GALNS activity in plasma and tissues was quantified as outlined in [36]. Frozen tissues were homogenized in a buffer consisting of 25 mM Tris-HCl (pH 7.2) and 1 mM phenylmethylsulphonyl fluoride, using an Omni Bead Ruptor Bead Mill Homogenizer (OMNI International, Kennesaw, GA, USA) at optimal speed and duration. The homogenate was transferred to a 1.5 mL tube and centrifuged for 30 min at 12,000× *g* at 4 °C. The resulting clear supernatant was used for enzyme activity measurement. Plasma or tissue lysates were incubated with 22 mM 4-methylumbelliferyl-β-galactopyranoside-6-sulfate (Carbosynth, San Diego, CA, USA) in 0.1 M NaCl/0.1 M sodium acetate (pH 4.3) at 37 °C in a HEIDOLPH incubator (Grainger, Lake Forest, IL, USA) for 17 h. Subsequently, 10 mg/mL β-galactosidase from *Aspergillus oryzae* (Sigma-Aldrich, St. Louis, MO, USA) in the same buffer was added, followed by incubation at 37 °C for an additional hour. The reaction was halted with 1 M glycine and NaOH (pH 10.5), and fluorescence was measured at excitation/emission wavelengths of 366/450 nm on a PerkinElmer Victor X4 plate reader (PerkinElmer, Waltham, MA, USA). Enzyme activity was expressed as nanomoles of 4-methylumbelliferone released per hour per milliliter of plasma or per milligram of protein. Protein concentration was determined using a bicinchoninic acid protein assay kit (Thermo Fisher Scientific, Waltham, MA, USA).

### 2.4. Glycosaminoglycans Assay

Keratan sulfate levels in blood and tissues were measured using liquid chromatography–tandem mass spectrometry (LC-MS/MS). Ten microliters of plasma or serum, or standard, along with 90 μL of 50 mM Tris-HCl (pH 7.0), were added to wells of an AcroPrep™ Advance 96-Well Filter Plate equipped with Ultrafiltration Omega 10 K membrane filters (PALL Corporation, Port Washington, NY, USA). Each well received a 40 μL cocktail containing recombinant chondroitinase B, heparitinase, and keratanase II (all at 1 mU per sample), plus IS solution (5 μg/mL), followed by 60 μL of 50 mM Tris-HCl (pH 7.0). The filter plate was placed on a 96-well receiving plate and incubated at 37 °C overnight, then centrifuged at 14.4× *g* for 20 min. The samples were injected into the LC-MS/MS system, consisting of a 1290 Infinity LC coupled to a 6460 triple quadrupole mass spectrometer (Agilent Technologies, Palo Alto, CA, USA). An injection volume of 5 μL was used, with a Hypercarb column (2.0 mm i.d., 50 mm length, 5 μm particle size; Thermo Fisher Scientific, Waltham, MA, USA) maintained at 60 °C for disaccharide separation. The mobile phases were 100 mM ammonia (A) and 100% acetonitrile (B). The gradient was programmed to start with 100% A for 1 min, gradually shifting to 30% B over 4 min, holding at 30% B for 5.5 min, then reverting to 0% B over 6 min, and maintaining 0% B until 10 min. The flow rate was set at 0.7 mL/min. Specific precursor and product ions (*m/z*) were used to quantify each disaccharide: IS, 354.3/193.1; DS, 458.4/300.2; mono-sulfated KS, 462/97; di-sulfated KS, 542/462; diHS-NS, 416/138; diHS-0S, 378.3/175.1. [36,53,54,55]. The concentration of each disaccharide was calculated using QQQ Quantitative Analysis (B.05.00/Build 5.0.291.0) software.

### 2.5. Glycosaminoglycan Extraction from Tissues

GAG extraction from tissue was performed as described previously [1,56], with minor modifications. Frozen mouse tissues (30–50 mg) were dissected and homogenized in cold acetone using homogenization tubes (2 mL microtubes with 2.8 mm ceramic beads) and an Omni Bead Ruptor Bead Mill Homogenizer (OMNI International, Kennesaw, GA, USA) at a suitable speed and duration. The tissue homogenate was transferred to a 1.5 mL tube and centrifuged for 30 min at 12,000× *g* at 4 °C. The acetone buffer was discarded, and the defatted pellets were dried in a vacuum centrifuge. To the dried pellet, 200 µL of 0.5 M NaOH was added, and the mixture was incubated for 2 h at 50 °C to cleave GAG chains from the core protein. After neutralizing with 1 N HCl (100 µL), NaCl was added to reach 3 M. The suspension was centrifuged again to remove nucleotides, and the supernatant’s pH was lowered below 1.0 with 1 N HCl (83.3 µL) to precipitate proteins. After centrifugation, the supernatant was neutralized with 1 N NaOH (83.3 µL). The crude GAGs were precipitated by adding twice the volume of ethanol containing 1.3% potassium acetate. Following centrifugation, the dried precipitate was dissolved in 50 mM Tris-HCl (pH 7.0).

### 2.6. Detection of Plasma Anti-GALNS IgG Antibodies

The presence of anti-hGALNS antibodies in plasma was measured using an indirect ELISA, as described earlier [35,57]. Briefly, 96-well plates were coated overnight with 2 μg/mL of purified rhGALNS (R&D Systems, Minneapolis, MN, USA) in a solution containing 15 mM Na_2_CO_3_, 35 mM NaHCO_3_, and 0.02% NaN_3_ at pH 9.6. The plates were then blocked for 1 h at room temperature with 3% bovine serum albumin in PBS (pH 7.2). Diluted plasma (1:100) was added to the wells and incubated at 37 °C for 2.5 h. A secondary antibody, peroxidase-conjugated goat anti-mouse IgG (Thermo Fisher Scientific, Waltham, MA, USA), was used at a 1:1000 dilution and incubated at room temperature for 1 h. Peroxidase substrate (ABTS, Invitrogen, Carlsbad, CA, USA) was added, and the plates were incubated for 30 min. The reaction was stopped with 1% SDS, and the absorbance was measured at 410 nm using a PerkinElmer Victor X4 plate reader (PerkinElmer, Waltham, MA, USA).

### 2.7. Quantification of AAV Genome Copies

AAV genome copies in the tissues (liver and bone) were quantified using the digital PCR method as previously described [2]. Tissues were processed into DNA using the Qiagen Puregene Kit (Germantown, MD, USA), including both Proteinase K and RNase treatments for all samples. DNAs were resuspended in Low TE, and DNA concentration was measured by both Nanodrop (ThermoFisher) and Qubit (Invitrogen). 550 ng of DNA (as determined by Qubit concentration) in a total volume of 55 μL was sheared to 800 bp using the Covaris M220 Focused-ultrasonicator in microTUBE-50 AFA Fiber Screw-Cap tubes.

To determine how close in range AAV Copy number was to Tfrc (2 copy control gene), a single 10 µL multiplex reaction was run on a QuantStudio 12K Flex Real-Time PCR System in a 384-well plate under normal cycling conditions: 5 µL of 2X TaqMan TaqPath ProAmp Master Mix, 0.5 µL of 20X AAV FAM Assay (18 µM forward primer, 18 µM reverse primer, 5 µM probe), 0.5 µL of 20X TaqMan™ Mouse Tfrc VIC Copy Number Reference Assay, 1 µL (10 ng) of sheared DNA, and 3 µL of Nuclease-free water.

Digital PCR (dPCR) was performed on a ThermoFisher QuantStudio 3D Platform using specific primers and probe sequences for RBG pA, which were as follows: forward primer, 5′-GCCAAAAATTATGGGGACAT-3′; reverse primer, 5′-ATTCCAACACACTATTGCAATG-3′; and probe, 5′-6FAM-ATGAAGCCCCTTGAGCATCTGACTTCT-QSY-3′. Samples were run in multiplex (AAV and Tfrc on the same chip) or singleplex (AAV and Tfrc on separate chips), depending on whether they were close enough in range, as determined by the qPCR QuantStudio 12K data. 16 µL reactions were prepared with 8 µL of 2X QuantStudio 3D Digital Master Mix v2, 0.8 µL of appropriate 20X assay(s), up to 50 ng of sheared DNA, and nuclease-free water. A QuantStudio 3D Digital PCR Chip v2 was loaded with 14.5 µL of the reaction mixture, and the chips were run on an Applied Biosystems ProFlex™ 2x Flat PCR System under standard 3D cycling conditions: 10 min hold at 96 °C, 39 cycles of 60 °C for 2 min and 98 °C for 30 s, followed by a 2 min hold at 60 °C, and a final hold at 25 °C. After cycling, the chips were scanned on the QuantStudio 3D PCR instrument. The chip scans were imported into ThermoFisher’s QuantStudio 3D AnalysisSuite Cloud Software (V2), cps/µL for both AAV and Tfrc were determined, and the average relative copy number of AAV was calculated by comparison to Tfrc (2-copy control gene).

### 2.8. Assessment of Bone Pathology

Toluidine blue staining of hearts and knee joints followed the method previously described [58]. Briefly, tissues (bones and hearts) from MPS IVA and WT mice at 24 and 48 weeks were collected to examine storage granules using light microscopy. The tissues were fixed in 2% paraformaldehyde and 4% glutaraldehyde in PBS, then post-fixed with osmium tetroxide and embedded in Spurr’s resin. Sections 0.5 µm thick, stained with toluidine blue, were analyzed. To evaluate chondrocyte size (vacuolization) in the growth plates of the femur or tibia, about 200 chondrocytes in the proliferative zone per mouse were measured using ImageJ (version 2). Pathological slides from treated and untreated MPS IVA and wild-type mice’s knee joints were examined to assess reductions in vacuolization and improvements in column structure of the growth plate. Storage material amounts and column disorganization levels were scored. Each slide was reviewed three times in a double-blind manner, and scores were averaged across different bone sections (growth plate, articular disc, meniscus, and ligament) for each mouse group. 

### 2.9. Micro-Computed Tomography Analysis of Femur

Micro-computed tomography (micro-CT) analysis of the mouse femur was performed as described previously [3]. The femurs of 24-week-old male mice and 48-week-old female mice after AAV vector injection were examined with the SkyScan 1275 system (Bruker, Billerica, MA, USA) at a source voltage of 80 kV and a current of 125 μA. Three-dimensional microstructural images were reconstructed using Bruker Skyscan 1275 system, NRecon: 2.0, CTAn: v1.21.1.0. SkyScan NRecon software. Bruker CTAN software was then used to determine trabecular bone volume (BV/TV, %), trabecular number (Tb.N, 1/mm), and bone mineral density (BMD, gHA/cm^3^).

### 2.10. Data Analysis

Statistical analysis was conducted using two-tailed unpaired Student’s *t*-tests and nonparametric tests with GraphPad Prism 9.0 (GraphPad, San Diego, CA, USA). Data are presented as means with standard deviations (SDs). Results with a *p*-value of less than 0.05 were deemed statistically significant.

## 3. Results

### 3.1. Enzyme Activity and KS Level in Plasma

#### 3.1.1. GALNS Enzyme Activity in Plasma

**Figure 1:**
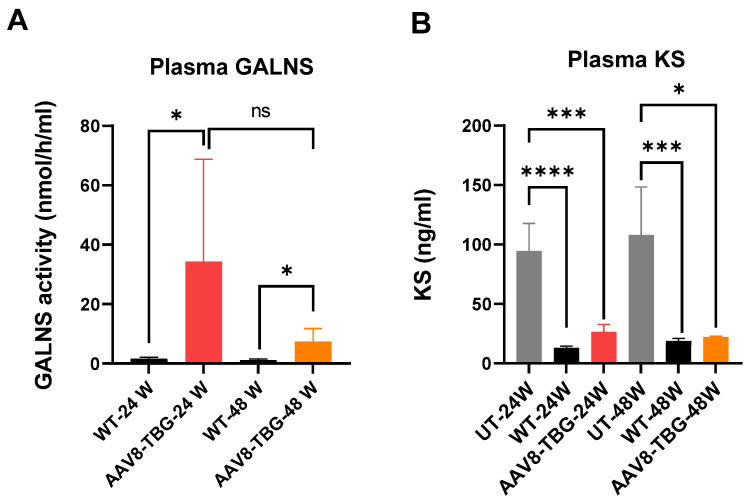
**Enzyme Activity and keratan sulfate level in plasma. Plasma GALNS activity** (**A**): WT versus AAV8-TBG (*t*-test, * *p* < 0.05) at 24 weeks. WT versus AAV8-TBG at 48 weeks (*t*-test, * *p* < 0.05). No significant difference was observed in plasma GALNS activity between the 24-week and 48-week treatment groups. **Plasma KS level** (**B**): At 24 weeks, UT versus WT (*t*-test, **** *p* < 0.0001), UT versus AAV8-TBG (*t*-test, *** *p* < 0.001). At 48 weeks, UT versus WT (*t*-test, *** *p* < 0.001), UT versus AAV8-TBG (*t*-test, * *p* < 0.05). No significant difference was found in plasma KS level between the 24-week and 48-week treatment groups. UT; untreated, WT; wild-type, TBG; thyroxin-binding globulin, ns; non-significant.

In this study, we evaluated the long-term (24–48 weeks) effects of an AAV vector expressing the human GALNS enzyme, driven by a liver-specific human thyroxin-binding globulin (TBG) promoter, in GALNS knockout (KO, *Galns^−/−^*) MPS IVA mouse models with a C57BL/6 background. Homozygous male and female MPS IVA mice at 4 weeks of age received an intravenous dose of 5 × 10^13^ GC/kg of AAV8-TBG-hGALNS via the lateral tail veins. Plasma GALNS activity results are shown in Figure 1. Male mice at 24 weeks exhibited significantly higher plasma activity (34.4 nmol/h/mL; *p* < 0.05) compared to WT mice (1.66 nmol/h/mL). Female mice at 48 weeks also showed significantly increased plasma activity (7.4 nmol/h/mL; *p* < 0.05) relative to WT mice (1.08 nmol/h/mL). Overall, plasma enzyme activity was higher in male mice compared to female mice (Figure 1A).

#### 3.1.2. Keratan Sulfate Levels in Plasma

KS levels in both 24-week and 48-week untreated mice were significantly higher than those in WT mice (*p* < 0.0001 and *p* < 0.001, respectively; Figure 1B). The KS level in 24-week-treated mice was significantly lower compared to untreated mice (*p* < 0.001). The KS level in 48-week-treated mice was brought back to the WT level (Figure 1B). In summary, plasma KS decreased in both 24-week and 48-week-treated mice.

### 3.2. GALNS Enzyme Activity in Tissues

#### 3.2.1. GALNS Enzyme Activity in Liver

**Figure 2:**
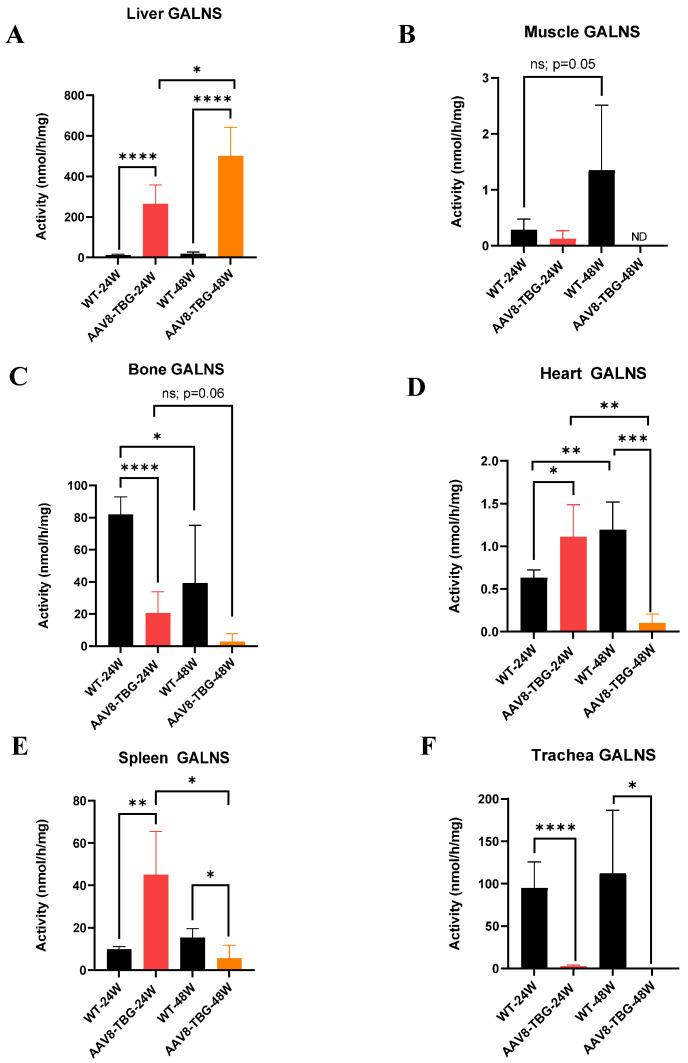
**Tissues GALNS activity at 24 and 48 weeks.** Liver GALNS activity (**A**): The AAV8-TBG group shows higher activity than the WT group at both 24 and 48 weeks (*t*-test, **** *p* < 0.0001). Activity at 48 weeks exceeds that at 24 weeks in the AAV8-TBG group (*t*-test, * *p* < 0.05). Muscle GALNS activity (**B**): The WT at 24 weeks has lower GALNS activity than at 48 weeks. Bone GALNS activity (**C**): GALNS activities in the AAV8-TBG group at 24 and 48 weeks are lower than those in WT (*t*-test, *** *p* < 0.0001 and not significant, respectively). Activity at 48 weeks is higher than at 24 weeks in the WT group (*t*-test, * *p* < 0.05). Heart GALNS activity (**D**): At 24 weeks, AAV8-TBG activity is significantly higher (*t*-test, * *p* < 0.05); it is also significantly higher at 24 weeks than at 48 weeks (*t*-test, ** *p* < 0.01). AAV8-TBG activity at 48 weeks is significantly lower than in WT (*t*-test, *** *p* < 0.001), and WT at 48 weeks is higher than at 24 weeks (*t*-test, ** *p* < 0.01). Spleen GALNS activity (**E**): At 24 weeks, AAV8-TBG is significantly higher than WT (*t*-test, ** *p* < 0.01), and at 24 weeks, AAV8-TBG is also significantly higher than at 48 weeks (*t*-test, * *p* < 0.05). AAV8-TBG activity at 48 weeks is significantly lower than in WT (*t*-test, * *p* < 0.05). Trachea GALNS activity (**F**): AAV8-TBG at both 24 and 48 weeks is significantly lower than in WT (*t*-test, **** *p* < 0.0001 and * *p* < 0.05, respectively). WT: wild-type, TBG: thyroxin-binding globulin, ns; non-significant.

Liver GALNS activity is shown in Figure 2A. In 24-week-treated male mice, the enzyme activity (264 nmol/h/mg) was significantly higher (*p* < 0.0001) than in WT mice (11 nmol/h/mg). In 48-week-treated female mice, the enzyme activity (500 nmol/h/mg) was significantly higher (*p* < 0.0001) than in WT mice (17 nmol/h/mg). The 48-week-old treated female mice had twice the GALNS activity compared to the 24-week-old treated male mice. The higher GALNS expression in female mice may be due to a longer treatment duration, leading to enzyme storage in the liver. The GALNS activity in untreated male mice was 0.03 nmol/h/mg, and it was undetectable in untreated female mice.

#### 3.2.2. GALNS Enzyme Activity in Muscle

Muscle GALNS activity is shown in Figure 2B. GALNS enzyme activity in 24-week-treated male mice was lower than in WT mice (0.13 and 0.28 nmol/h/mg, respectively). In 48-week-treated female mice, the enzyme activity was not detected. The study demonstrated that liver-specific promoters do not deliver enzymes to muscle. GALNS activity in both male and female untreated mice was not detected.

#### 3.2.3. GALNS Enzyme Activity in Bone

In bone, GALNS enzyme activity in 24-week-treated male mice (21 nmol/h/mg) was significantly lower than in 24-week-old WT mice (82 nmol/h/mg) (Figure 2C). The GALNS activity at 48 weeks in treated female mice was 2.8 nmol/h/mg, which is even lower than that in 24-week-old male mice. Additionally, the activity in 48-week-old WT mice (27 nmol/h/mg) was significantly lower than in 24-week-old male WT mice. Although liver GALNS activity was higher (500 nmol/h/mg) in female mice, the enzyme was not delivered sufficiently to the cartilage tissue. The GALNS activity in both untreated male and female mice was not detected.

#### 3.2.4. GALNS Enzyme Activity in the Heart

Heart GALNS activity is shown in Figure 2D. The GALNS enzyme activity at 24 weeks in treated male mice was significantly higher (1.1 nmol/h/mg; *p* < 0.05) than in WT mice (0.63 nmol/h/mg). At 48 weeks, the enzyme activity in treated female mice was significantly lower (0.01 nmol/h/mg; *p* < 0.001) than in WT mice (1.2 nmol/h/mg). Additionally, the activity of mice treated for 24 weeks was significantly higher (*p* < 0.01) than that of mice treated for 48 weeks. The GALNS activity in both untreated male and female mice was not detected.

#### 3.2.5. GALNS Enzyme Activity in Spleen

Spleen GALNS activity is shown in Figure 2E. GALNS enzyme activity at 24 weeks was significantly higher (*p* < 0.01) than that of WT, which measured 45 and 10 nmol/h/mg, respectively. At 48 weeks, GALNS enzyme activity was significantly lower (*p* < 0.05) than that of WT, with levels at 6 and 15 nmol/h/mg, respectively. Male mice exhibited significantly higher GALNS activity at 24 weeks (*p* < 0.05) compared to female mice treated for 48 weeks. These data suggest that enzyme activity in male mice was higher across most tissues (except the liver) than in female mice. GALNS activity was not detected in untreated male and female mice.

#### 3.2.6. GALNS Enzyme Activity in the Trachea

The GALNS enzyme activities in WT mice at 24 and 48 weeks were 95 and 112 nmol/h/mg, respectively, as shown in Figure 2F. In contrast, mice treated for 24 and 48 weeks had enzyme activities of 2.5 and 0.13 nmol/h/mg, respectively. The GALNS activity in both male and female untreated mice was undetectable.

Overall, GALNS activity was significantly higher than in other tissues, as shown in Figure 2.

### 3.3. KS Levels in Tissues

#### 3.3.1. KS Level in the Liver

**Figure 3:**
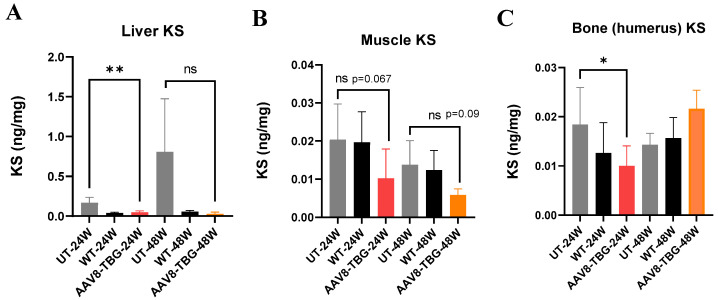
**Tissues KS at 24 and 48 weeks.** Liver KS (**A**): At 24 weeks, the KS level treated with AAV8-TBG was significantly lower than that with UT (*t*-test, ** *p* < 0.01). At 48 weeks, the KS level treated with AAV8-TBG was lower than that with UT; however, the difference was not statistically significant. Muscle KS (**B**): KS levels in both 24- and 48-week-treated mice were lower than those in the UT group, but the difference was not statistically significant. Bone KS (**C**): KS levels in bone (humerus) were significantly lower than those in UT (*t*-test, * *p* < 0.05) only at 24 weeks and were not affected at 48 weeks—UT; untreated, TBG; thyroxin-binding globulin, ns; non-significant.

KS levels in the liver of treated male mice at 24 weeks were significantly lower (0.05 ng/mg; *p* < 0.01) than in untreated mice (0.17 ng/mg) and normalized to WT levels (0.04 ng/mg) (Figure 3A). In treated female mice at 48 weeks, KS levels (0.027 ng/mg) were lower than in untreated mice (0.95 ng/mg) and normalized to WT levels.

#### 3.3.2. KS Level in Muscle

The KS level in the muscle of male mice was lower than in untreated mice, but the difference was not statistically significant (*p* = 0.067) (Figure 3B). Although GALNS activity in the muscle was not detected at 48 weeks in treated female mice, KS levels were reduced compared to untreated mice; however, this difference was not statistically significant (*p* = 0.09), indicating cross-correction.

#### 3.3.3. KS Level in Bone (Humerus)

KS level in the humerus of 24-week-old male mice was significantly reduced (*p* < 0.05) and normalized to WT levels (Figure 3C). Humerus KS level was not affected in 48-week-treated female mice. The most significant correction of KS was observed in the liver of both male and female treated mice.

### 3.4. Biodistribution of AAV Genome

Genome copies of AAV8 vectors in the liver and bone were quantified by digital PCR, as described in the Materials and Methods. In the liver of male mice treated with the AAV8 vector, the AAV8 vector was found in abundance (Figure S1A). At 48 weeks, the AAV8 vector was significantly higher (* *p* < 0.05) in treated female mice compared to untreated mice (Figure S1A). In 24-week-treated male mice, the AAV8 copy number in bone was higher than in 48-week-treated female mice (Figure S1B). Overall, the AAV8 copy number in bone was significantly lower than in the liver.

### 3.5. Humoral Response Against GALNS

**Figure 4:**
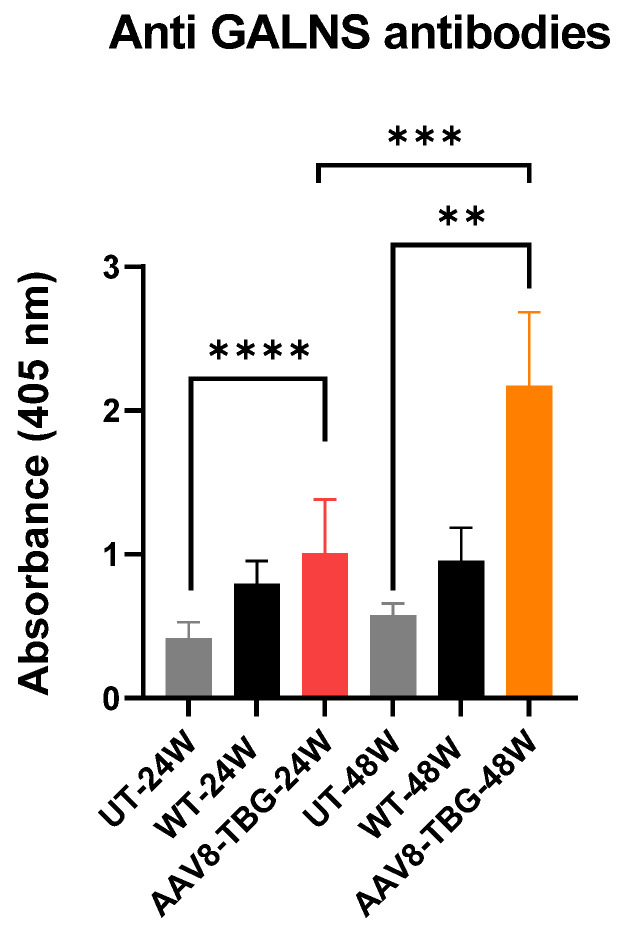
**Plasma anti-GALNS antibodies at 24 and 48 weeks.** The plasma GALNS antibodies in both 24- and 48-week AAV8-TBG-treated mice were significantly higher than in their UT mice (*t*-test, **** *p* < 0.0001 and ** *p* < 0.01, respectively). Anti-GALNS antibodies in AAV8-TBG-treated mice at 48 weeks were also significantly higher than those in 24-week AAV8-TBG-treated mice (*t*-test, *** *p* < 0.001) UT; untreated, TBG; thyroxin-binding globulin.

Anti-hGALNS antibodies were measured in the plasma of WT, untreated, and treated mice using enzyme-linked immunosorbent assay (ELISA) (Figure 4). Both male and female treated groups had significantly higher levels of circulating anti-hGALNS antibodies than untreated mice (*p* < 0.0001 and <0.01, respectively). The level of anti-GALNS antibodies was significantly higher (*p* < 0.001) in 48-week-treated female mice than in 24-week-treated male mice (Figure 4).

### 3.6. Micro-Computed Tomography Analysis of the Femur

**Figure 5:**
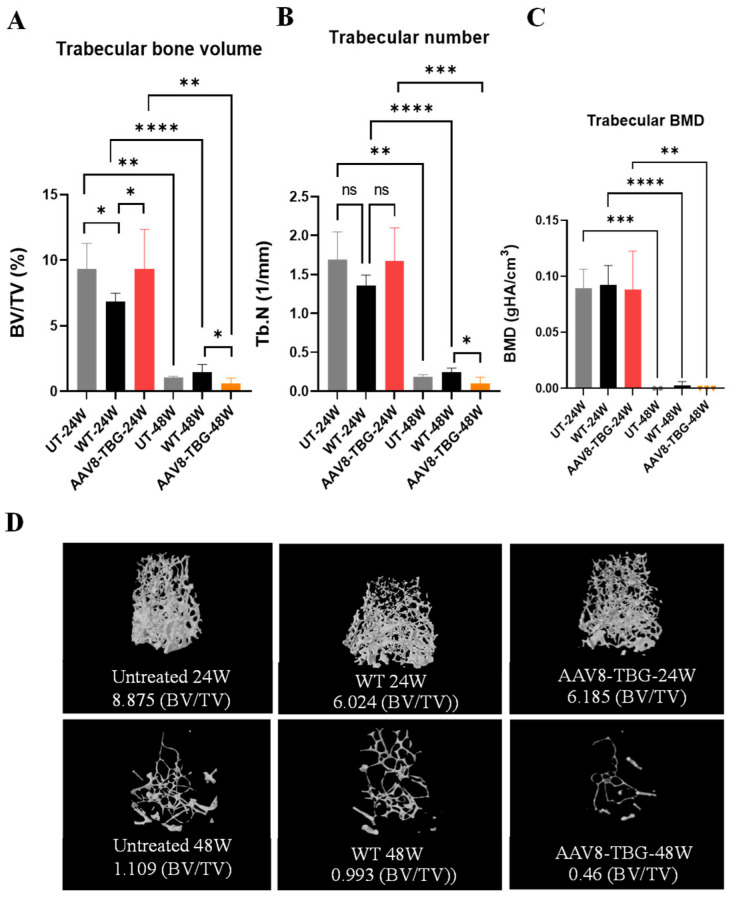
**Trabecular Bone Morphometry.** Trabecular bone volume fraction (**A**): Trabecular bone volume: 24 weeks; UT versus WT (* *p* < 0.05), WT versus AAV8-TBG (* *p* < 0.05). 48 weeks; WT versus AAV8-TBG (* *p* < 0.05). Comparison of 24 and 48 weeks within groups: UT (*t*-test, ** *p* < 0.01), WT (*t*-test, **** *p* < 0.0001), AAV8-TBG (*t*-test, ** *p* < 0.01). One-way ANOVA, Kruskal–Wallis test. Trabecular number (**B**): Trabecular number; at 48 weeks: WT versus AAV8-TBG (* *p* < 0.05). 24 weeks versus 48 weeks in UT (*t*-test, ** *p* < 0.01), WT (*t*-test, **** *p* < 0.0001), and AAV8-TBG (*t*-test, *** *p* < 0.001). Trabecular BMD (**C**): Trabecular BMD; at 24 weeks: UT versus 48 weeks (*t*-test, *** *p* < 0.001). 24 weeks versus 48 weeks in WT (*t*-test, **** *p* < 0.0001), and AAV8-TBG (*t*-test, ** *p* < 0.01). Trabecular bone structure (BV/TV) at 24 weeks for UT, WT, and AAV8-TBG (upper panel), and at 48 weeks for UT, WT, and AAV8-TBG (lower panel) (**D**). Statistical analyses included one-way ANOVA and Kruskal–Wallis tests. UT; untreated, WT; wild-type, TBG; thyroxin-binding globulin, ns; non-significant.

Micro-computed tomography (micro-CT) analysis of mouse femurs was performed. Trabecular bone morphometry is shown in Figure 5A–C. Trabecular bone volumes in 24-week-treated male mice did not change compared to those of untreated mice (24 weeks). However, 24-week WT mice were significantly lower (*p* < 0.05) than 24-week untreated mice (Figure 5A). Trabecular bone volumes in 48-week treated female mice were lower than those of untreated mice, though not significantly. Additionally, the 48-week untreated, WT, and treated groups were significantly lower than the 24-week groups (*p* < 0.01, <0.0001, and <0.01, respectively). Trabecular numbers in 24-week-treated male mice were not changed compared to untreated mice; however, WT was lower than that of untreated mice but not significantly (Figure 5B). Trabecular numbers in 48-week-treated female mice were lower than those of untreated mice but not significantly. Moreover, the 48-week untreated, WT, and treated groups were significantly lower than the 24-week groups (*p* < 0.01, <0.0001, and <0.001, respectively). Bone mineral density (BMD) in both 24- and 48-week groups was not lower than that of their untreated groups (Figure 5C). However, 48-week untreated, WT, and treated groups were significantly lower than the 24-week groups (*p* < 0.001, <0.0001, and <0.01, respectively). Additionally, the 48-week female groups showed significantly lower values compared to the 24-week male mouse groups. Micro-CT trabecular bone (percent bone volume) [BV/TV; bone volume/total volume (bone + other tissues)] of both male and female treated groups was reduced compared to their untreated mice (Figure 5D).

**Figure 6:**
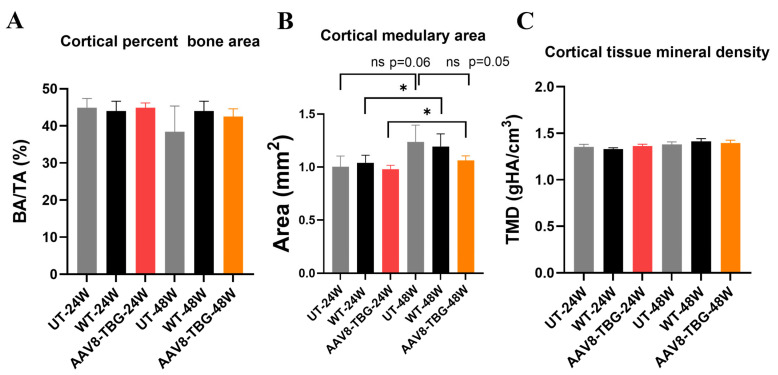
**Cortical bone architecture.** Cortical percent bone area (**A**): No significant differences are shown among UT, WT, and AAV8-TBG-treated mice at 24 and 48 weeks. Cortical medullary area (**B**): Significant changes are demonstrated in WT between 24 weeks and 48 weeks (* *p* < 0.05); also, in AAV8-TBG between 24 weeks and 48 weeks (* *p* < 0.05). Cortical tissue mineral density (**C**): No significant differences are shown among UT, WT, and AAV8-TBG-treated mice at 24 and 48 weeks—UT; untreated, WT; wild-type, TBG; thyroxin-binding globulin, ns; non-significant.

The cortical bone architecture was analyzed and shown in Figure 6A–C. Cortical bone areas and cortical tissue mineral density (TMD) in both male and female treated groups remained unchanged (Figure 6A,C). The cortical medullary areas of both male and female groups remained unchanged. However, the cortical medullary area of the treated female group was significantly larger (*p* < 0.05) compared to the treated male mice group (Figure 6C).

### 3.7. Bone and Cartilage Pathology

**Figure 7:**
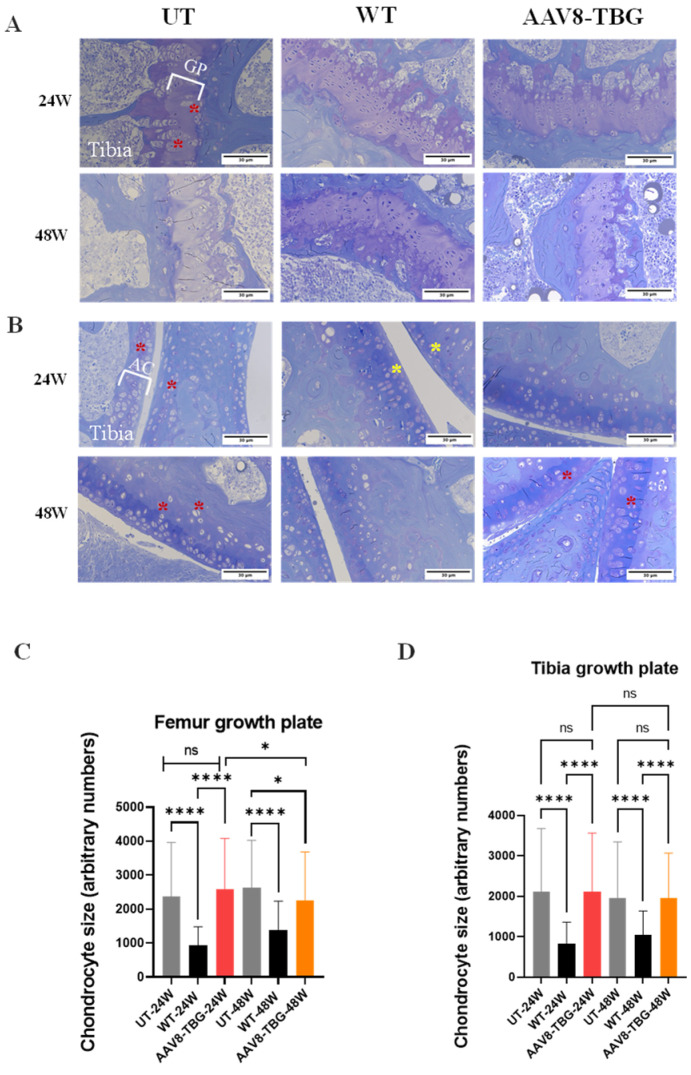
**Bone pathology (Knee joint).** Growth plate region (**A**): Top panel shows 24 weeks UT, WT, and AAV8-TBG; bottom panel shows 48 weeks UT, WT, and AAV8-TBG. Articular cartilage region (**B**): Top panel shows 24 weeks UT, WT, and AAV8-TBG; bottom panel shows 48 weeks UT, WT, and AAV8-TBG. Chondrocytes (200–300 per group) and cell size in growth plate lesions of the femur (**C**) and tibia (**D**) were analyzed. Yellow asterisks mark representative non-vacuolated chondrocytes; red asterisks mark vacuolated chondrocytes. Chondrocyte size was measured using ImageJ. Statistical analyses included one-way ANOVA and Kruskal–Wallis tests. Femur: at 24 weeks, UT versus WT (**** *p* < 0.0001); at 48 weeks, UT versus WT (**** *p* < 0.0001) and versus AAV8-TBG (* *p* < 0.05). Tibia: UT versus WT at both 24 and 48 weeks (**** *p* < 0.0001). Differences between UT and AAV8-TBG at both points were not significant. UT; untreated, WT; wild-type, TBG; thyroxin-binding globulin, GP; Growth plate, AC; Articular cartilage, ns; non-significant.

Previous studies assessed the impact of AAV8-TBG on the femurs and tibias of MPS IVA mice at 16 weeks. In untreated male mice at 24 weeks, marked GAG storage vacuolation was evident in the tibia growth plates, articular cartilage, meniscus, and ligaments (Figure 7A,B). These growth plates exhibited disorganized column structures with vacuolated chondrocytes. In the 24-week AAV8-TBG-treated groups, there was a partial reduction in storage material across the growth plates, articular cartilage, ligaments, and knee menisci. While the chondrocyte column structure improved, it remained disorganized and distorted (Figure 7A,B). Untreated female mice at 48 weeks exhibited even more GAG storage vacuoles in the same tissues, characterized by disorganized column structures and vacuolated chondrocytes (Figure 7A,B). By 48 weeks, females treated with AAV8-TBG displayed a less pronounced reduction in storage material than the 24-week males, and their chondrocyte column structure remained highly disorganized and distorted.

The size of chondrocyte cells was measured to evaluate the improvement in vacuolization of cartilage cells in the growth plate. Chondrocyte cell size was significantly decreased in the growth plate lesions of femurs in the 48-week treated (AAV8-TBG) group. However, the 24-week-treated group did not show a reduction in chondrocyte cell size in the femur (Figure 7C). Tibia growth plate chondrocytes were unaffected in both the 24-week and 48-week treated groups. (Figure 7D).

**Figure 8:**
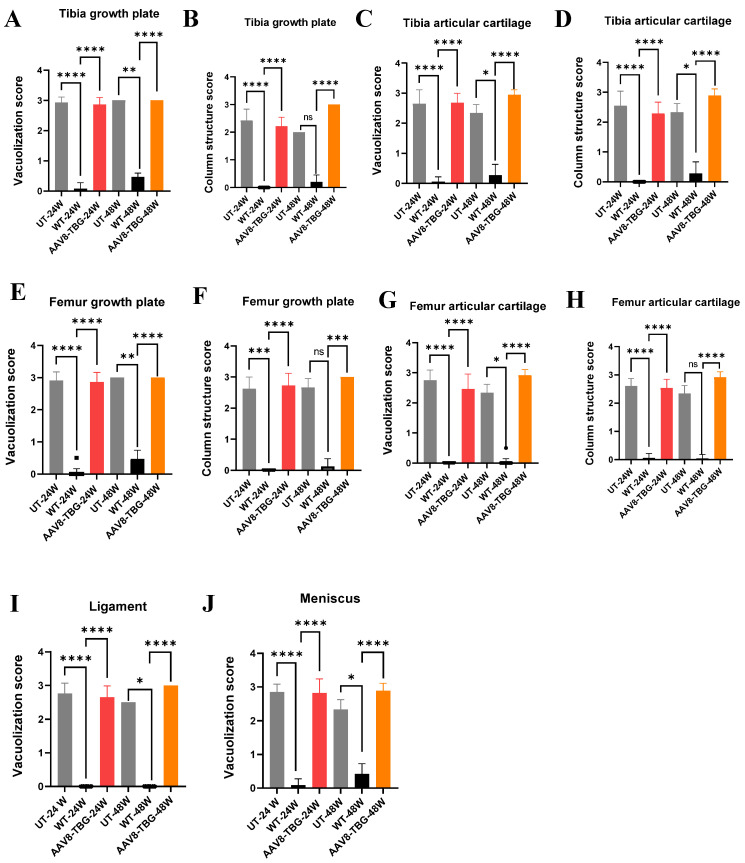
Tibia and Femur growth plate and articular cartilage score for vacuolization and column structure (**A**–**H**). Ligament and Meniscus vacuolization (**I**,**J**). Tibia growth plate vacuolization score (**A**): One-way ANOVA, Kruskal–Wallis test. Both 24- and 48-week UT versus WT (**** *p* < 0.0001 and ** *p* < 0.01, respectively). Both 24- and 48-week WT versus AAV8-TBG (**** *p* < 0.0001). Tibia growth plate column structure score (**B**): 24-week UT versus WT and WT versus AAV8-TBG (**** *p* < 0.0001). 48-week WT versus AAV8-TBG (*** *p* < 0.0001). Tibia articular cartilage vacuolization score (**C**): Both 24- and 48-week UT versus WT (**** *p* < 0.0001 and * *p* < 0.05, respectively). Both 24- and 48-week WT versus AAV8-TBG (**** *p* < 0.0001). Tibia articular cartilage column structure score (**D**): Both 24- and 48-week UT versus WT (**** *p* < 0.0001 and * *p* < 0.05, respectively). Both 24- and 48-week WT versus AAV8-TBG (**** *p* < 0.0001). Femur growth plate vacuolization score (**E**): Both 24- and 48-week UT versus WT (**** *p* < 0.0001 and ** *p* < 0.01, respectively). Both 24- and 48-week WT versus AAV8-TBG (**** *p* < 0.0001). Femur growth plate column structure score (**F**): 24-week UT versus WT versus AAV8-TBG (*** *p* < 0.001, and **** *p* < 0.0001, respectively). 48-week WT versus AAV8-TBG (*** *p* < 0.001). Femur articular cartilage vacuolization score (**G**): Both 24- and 48-week UT versus WT (**** *p* < 0.0001 and * *p* < 0.05, respectively). Both 24- and 48-week WT versus AAV8-TBG (**** *p* < 0.0001). UT versus WT (**** *p* < 0.0001). Femur articular cartilage column structure score (**H**): Both 24-week UT versus WT versus AAV8-TBG (**** *p* < 0.0001). 48 weeks, WT versus AAV8-TBG (**** *p* < 0.0001). Ligament vacuolization (**I**): Both 24- and 48-week UT versus WT (**** *p* < 0.0001 and * *p* < 0.05, respectively). Both 24- and 48-week WT versus AAV8-TBG (**** *p* < 0.0001). Meniscus vacuolization (**J**): Both 24- and 48-week UT versus WT (**** *p* < 0.0001 and * *p* < 0.05, respectively). Both 24- and 48-week WT versus AAV8-TBG (**** *p* < 0.0001). UT; untreated, WT; wild-type, TBG; thyroxin-binding globulin, ns; non-significant.

We also evaluated the improvement of vacuoles and disorganized column structures in MPS IVA mice using pathological scores (Figure 8A–J). These scores showed slight improvement in 24-week-treated male mice compared to untreated ones; however, pathological scores in 48-week-treated female mice remained unchanged (Figure 8A–J). Additionally, we assessed heart pathology. Both 24-week and 48-week untreated MPS IVA mice displayed GAG storage vacuoles in heart valves and muscles. Treated male mice showed moderate clearance of vacuoles; however, treated female mice showed no improvement in vacuole clearance (Supplemental Figure S2).

## 4. Discussion

In the present study, we aimed to investigate the long-term (24–48 weeks) impact and adverse effects of AAV gene therapy on MPS IVA mice using the liver-specific promoter (TBG). We have demonstrated that a single intravenous injection of the AAV8 vector encoding GALNS successfully establishes the liver as a bioreactor for continuous, systemic enzyme secretion. This strategy represents a significant conceptual advancement over existing therapies like ERT and HSCT, which have a limited impact on debilitating skeletal dysplasia [4,5,59,60]. The long-term potential of this strategy is the achievement of sustained, supraphysiological levels of GALNS activity in the plasma of both male (24 weeks) and female (48 weeks) mice, with a significant reduction in KS levels approaching the wild-type level. Due to TBG driving massive overexpression, successful transduction of hepatocytes was evident, with supraphysiological levels of GALNS activity in the livers of both treated groups; however, in the female-treated group, activity was almost 2-fold higher than in the male group, resulting in KS removal in both groups below WT levels. The GALNS enzyme activities in muscle, heart, bone, and trachea were low due to the liver-specific promoter. No serious adverse effect was observed during the long-term study.

The durability of the AAV-mediated approach stems from the stable, non-integrating episomal concatemers within the nuclei of long-lived hepatocytes [6,7,61,62]. Furthermore, our previous study, which lasted 16 weeks, demonstrated the efficacy of AAV gene therapy for MPS IVA mice using both tissue-specific and ubiquitous promoters, resulting in a significant improvement in biochemical parameters, normalization of KS levels, and a reduction in vacuoles in the heart muscle and valves, as well as in bone [8]. The study demonstrated higher enzyme activity in the heart and muscle following the activation of the liver–muscle tandem promoter. Similarly, ubiquitous and liver–bone tandem promoters had better enzyme activity in bone. In comparison to our previous study, we did not observe a significant change in bone morphology using micro-CT.

In this study, treated male mice exhibited GALNS levels in the liver that exceeded the normal physiological range, as indicated by increased plasma and organ levels. Although female mice had liver GALNS expression twice that of males, their plasma and organ GALNS levels were notably lower. Female mice demonstrated reduced treatment effectiveness, indicated by higher antibody production (Figure 4), decreased GALNS activity in plasma, and higher KS concentrations in bone relative to males. These results highlight the importance of sex in influencing therapeutic outcomes in this model. They also corroborate our earlier findings of a strong antibody response in females, which led to decreased GALNS enzyme activity and less effective tissue therapy compared to males [9].

The study found that female mice had antibody titers about 4.6 times higher than males, while GALNS enzyme activity was roughly 6.8 times lower in females [9]. Male mice receiving AAV-based gene therapy showed significant improvement in the femur and tibial growth plates, ligaments, and articular cartilage, as indicated by differences in pathology scores when compared to females. Cardiac histology demonstrated that vacuolation was not normalized in females, whereas in males, it was fully corrected [9].

This observed sexual dimorphism is well supported by the principle that females usually mount stronger innate and adaptive immune responses than males, as shown by both preclinical and broader immunological research [63]. The mechanisms are complex, involving hormonal and genetic influences [63,64]. Sex hormones are important modulators of immunity; estrogens are potent enhancers of humoral immunity, promoting B-cell activation and antibody production, while androgens, such as testosterone, are generally considered immunosuppressive [65]. This provides a direct hormonal explanation for the higher antibody response seen in female mice. Besides hormonal factors, genetics also plays a crucial role. The X chromosome contains several immune-related genes, some of which escape X-chromosome inactivation in females [64]. A key example is Toll-like receptor 7 (*TLR7*), an innate immune sensor that recognizes single-stranded viral genomes [66,67]. Since *TLR7* escapes inactivation, female immune cells can have a higher effective gene dose, resulting in a lower activation threshold and a stronger initial innate immune response to the AAV vector, which can then enhance the adaptive immune response against the transgene product [67,68]. The clinical importance of these sex-based differences is also becoming clearer, with studies in other areas showing variations in host inflammatory responses to AAV gene therapy by age and sex [66,67,68].

Various strategies have been used to counteract the immune response, including liver targeting to induce immune tolerance [10,11] and the use of steroids to suppress T cell responses against the capsid, ensuring long-lasting transgene expression [11,12]. These are some of the most common methods seen in clinical and preclinical studies [13,14]. Designing vectors that target the liver leverages the organ’s tolerogenic environment, thereby reducing the immune response triggered by the body. AAV8 is preferred for liver-directed approaches due to its high efficiency in transducing hepatocytes compared to other AAV serotypes, with transduction rates in mouse models ranging from 90% to 95%, depending on the dose [6]. Moreover, using liver-specific promoters can further enhance hepatocyte targeting and minimize transgene expression in non-target tissues. transgene production [6].

The safety of this approach at the administered dose is supported by extensive clinical experience with AAV8 vectors in human trials, particularly for hemophilia [15,69,70,71,72,73,74,75]. The most common adverse event observed in those trials is a dose-dependent, immune-mediated hepatotoxicity, which presents as a transient elevation in liver transaminases [15,16]. This reaction is now well-understood to be a T-cell response against the AAV capsid and is effectively managed with a tapering course of corticosteroids, which preserves the transduced liver cells and ensures long-term therapeutic benefit [16,17]. While the overall safety profile is favorable, a comprehensive assessment must also address the theoretical risk of insertional mutagenesis. Although AAV vectors persist primarily as non-integrating episomes, low-frequency integration into the host genome can occur [18,19,20]. Long-term studies in large animal models, such as a 10-year follow-up of dogs with hemophilia A treated with liver-directed AAV, have shown that while vector integration and subsequent benign clonal expansion of hepatocytes can occur, this process did not lead to the development of liver cancer or other adverse clinical outcomes [18,21]. However, the risk of genotoxicity can be influenced by factors such as vector dose and the specific promoter and enhancer elements used, as has been demonstrated in sensitive mouse models [18,19]. Therefore, in alignment with best practices for preclinical development, future studies should incorporate rigorous safety assessments, including performing genome sequencing and off-target studies to exclude potential off-target toxic effects.

These methods, when combined, have demonstrated the potential of AAV-mediated gene therapy in treating MPS IVA and other inherited metabolic disorders.

Recombinant AAV vectors are currently being extensively studied in numerous clinical trials and have shown great promise for human gene therapy. Their effectiveness is exemplified by the approval of six gene therapy products based on wild-type AAV serotypes: Glybera (AAV1), Luxturna (AAV2), and, most recently, Zolgensma (AAV9) [76], as well as Valoctocogene roxaparvovec (AAV5) for hemophilia A [77], and AAV8 and AAV9 vectors for hemophilia B [78]. The rationale behind this therapy, which aims for long-term benefits, hinges on how AAV vectors function in the liver. After administration, the AAV genome forms stable, circular episomes within the nucleus of transduced hepatocytes. Since hepatocytes are long-lived with a low turnover rate, these episomes can serve as lasting templates for transgene expression for many years [79,80]. Strong evidence for this long-term durability comes from landmark clinical trials for hemophilia B [66,81]. One study using a liver-targeted AAV8 vector demonstrated stable Factor IX expression, resulting in sustained clinical benefits and a significant reduction in bleeding rates over a median follow-up of 13 years following a single infusion [66]. This extensive clinical data underscores that a single dose of a liver-targeted AAV vector can create a durable biofactory for ongoing, long-term therapeutic protein production [66,81]. The number of clinical trials involving AAV-based gene therapies is expected to continue to grow, surpassing 200 by 2022 [82]. In the MPS field, the TBG promoter has been shown to enhance bone length in MPS VI feline and rat models [83,84], leading to ongoing clinical trials for MPS VI using a liver-specific promoter in an AAV8 vector (NCT03173521) [85]. Wood and Bigger provide an overview of gene therapy clinical trials for MPS [86]. Different promoters, such as CAG, have been used in clinical trials for MPS I, II, and IIIA, administered via various routes, demonstrating therapeutic effects (NCT03580083, NCT03566043, NCT03612869). Additionally, Yang et al. found that different AAV serotypes show varying efficiencies in transducing bone cells, including osteoblasts and chondrogenic cells [87]. Chen et al. demonstrated that intra-articular injections of AAV2 achieve high transduction efficiency in chondrocytes located in the deep layer of articular cartilage [88].

Despite the progress in AAV gene therapy, current AAV vectors need improvements in transduction efficiency, antibody escape, and tissue targeting to minimize dosages and enhance safety [22]. Consequently, further research should focus on optimizing AAV administration methods and selecting appropriate serotypes for treating bone and cartilage issues in MPS IVA mouse models and larger animals [2,9]. Since the TBG vector used here cannot fully resolve bone problems, employing a humanized mouse or rat model may better demonstrate the therapeutic potential of human GALNS cDNA.

Overall, previous studies on MPS IVA mice have examined the therapeutic effectiveness and side effects of AAV vectors with various promoters, including sex differences, in 16-week-old MPS IVA mice (mid-term) started at 4 weeks of age. In this study, we evaluated therapeutic effectiveness over a more extended period, adjusting the study duration accordingly. Female mice were selected at 48 weeks because we previously observed lower enzyme activity and higher anti-GALNS antibody levels in females compared to males. Conversely, male mice were selected at 24 weeks because they exhibited higher enzyme activity and lower anti-GALNS antibody levels. It was important to monitor long-term side effects in female mice. The issue of sex differences is discussed above. Importantly, data from both previous and current studies helped secure a clinical trial fund provided by the Foundation for the National Institutes of Health (FNIH). FNIH, along with 35 public, private, and nonprofit partners, established the Bespoke Gene Therapy Consortium (BGTC), which has selected and sponsored eight rare disorders, including Morquio A, to advance clinical trials [89,90].

The limitations of the current studies stem from the small number of mice; however, an apparent sex-related effect is evident, and therapeutic benefits are observed. To prevent the immune response against the GALNS enzyme, we are exploring methods to induce immune tolerance to the gene product in MPS IVA mice before AAV gene therapy (in submission). This immune tolerance, combined with AAV gene therapy, will enhance therapeutic effectiveness.

In conclusion, administering AAV8 intravenously with a liver-specific promoter provides a continuous supply of enzymes, resulting in sustained supraphysiological enzyme activity and improved therapeutic effectiveness—a crucial step toward advancing to clinical trials. However, additional improvements are necessary to address bone pathology and the immune response.

## Data Availability

Any raw data generated in this study is available upon request.

## Supplementary Materials

The following supporting information can be downloaded at: https://www.mdpi.com/article/10.3390/cimb47110900/s1.
