# Supplementary material for: Long-Term Liver-Targeted AAV8 Gene Therapy for Mucopolysaccharidosis IVA
Source: Curr Issues Mol Biol. 2025 Oct 29;47(11):900. doi: 10.3390/cimb47110900 (PMC12651378; doi:10.3390/cimb47110900)
Supplement: Supplementary file 1 [file cimb-47-00900-s001.zip › cimb-3899639-supplementary.pdf]

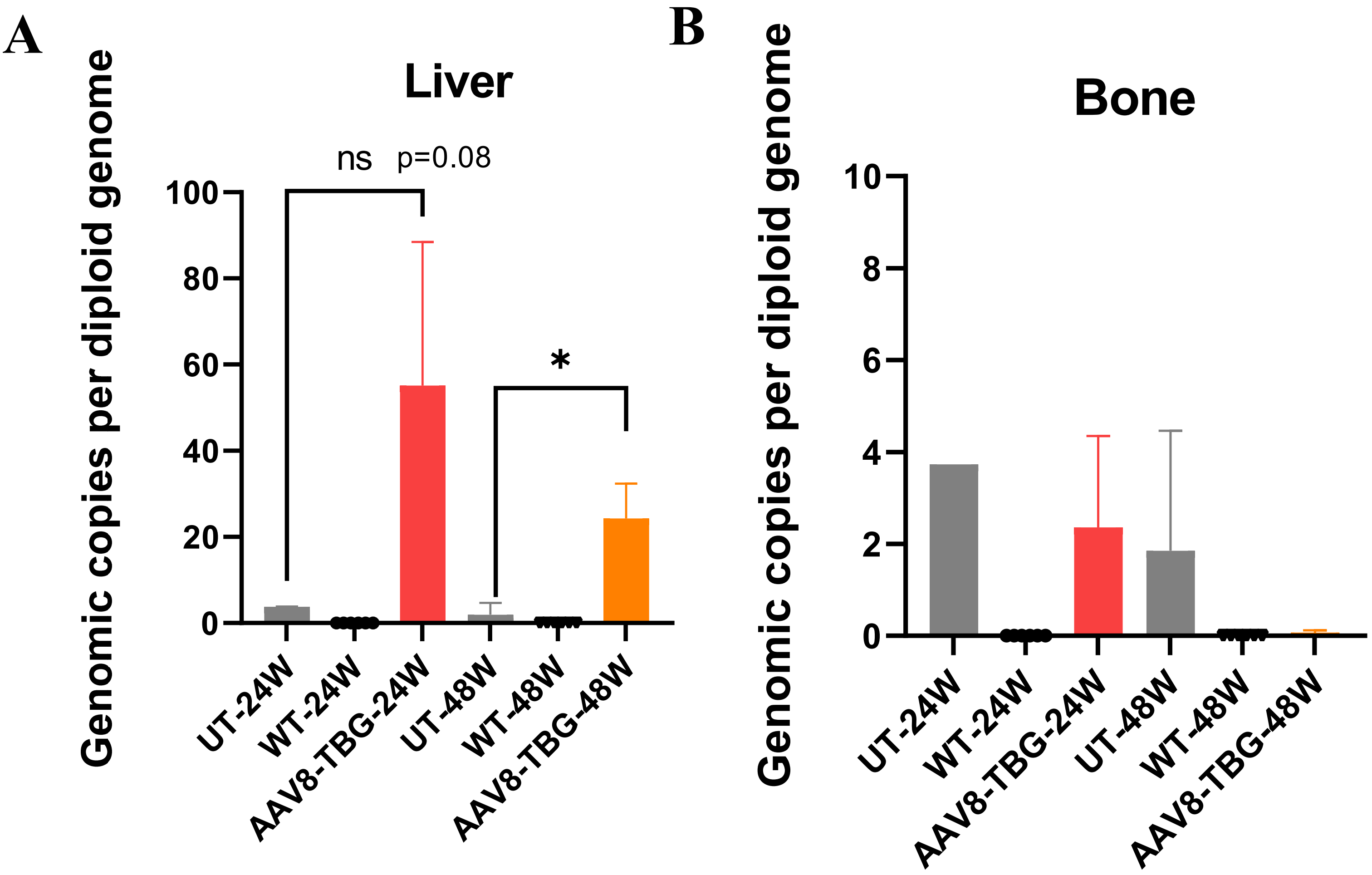

Figure S1. Tissue genomic copies per diploid genome. Liver (A); 24 weeks UT versus AAV8-TBG (*t*-test, $p = 0.08$). 48 weeks UT versus AAV8-TBG (*t*-test *$p < 0.05$). Bone (B); No statistical significance. UT; untreated, WT; wild-type, TBG; thyroxin-binding globulin, ns; non-specific.

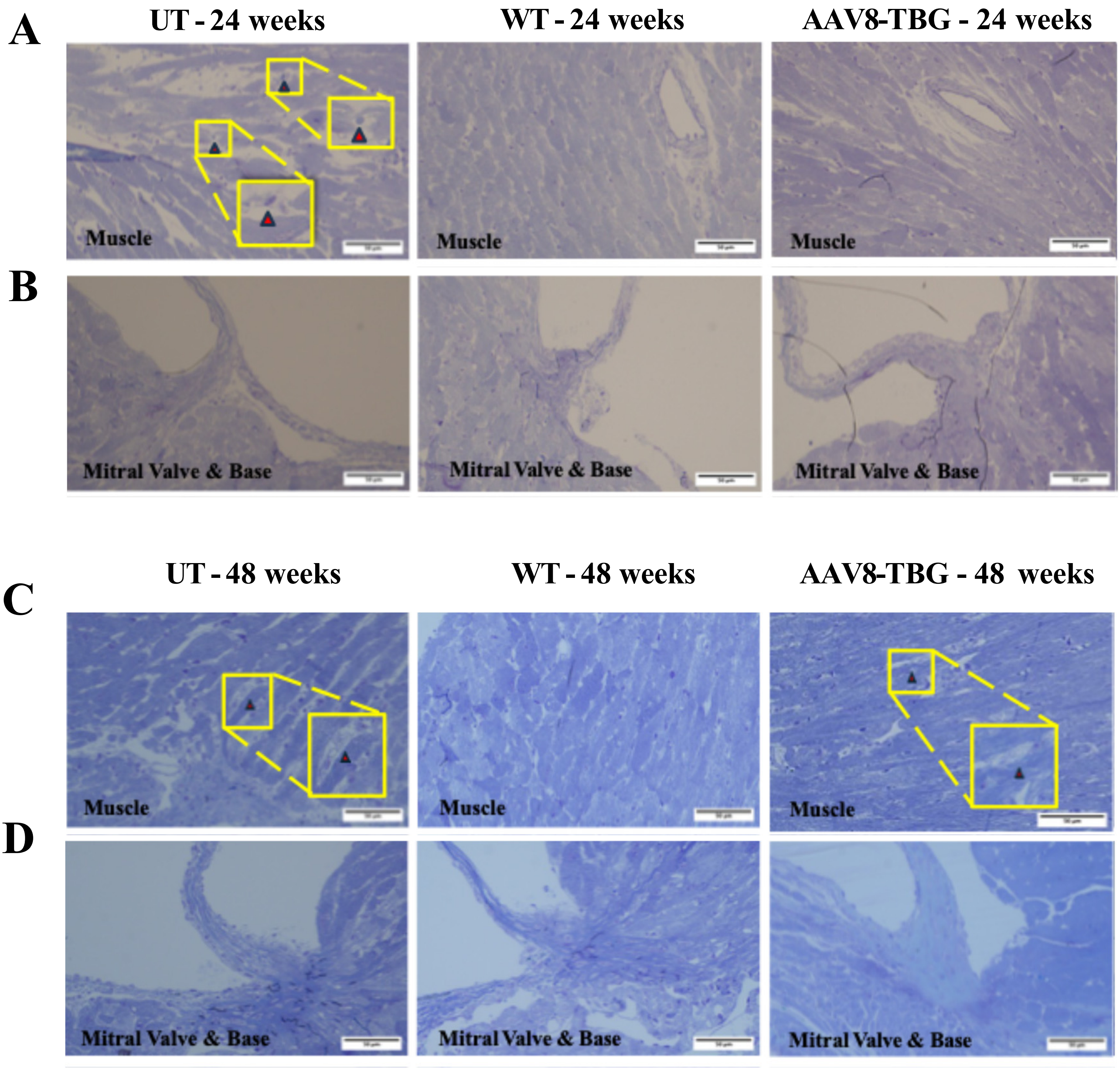

Figure S2. Heart pathology. Muscle (A), Valve (B), in **24 weeks** UT, WT, and AAV8-TBG. **Muscle (C), Valve (D),** in **48 weeks** UT, WT, AAV8-TBG. Red triangles demonstrate cardiomyocytes with vacuolar degeneration. The boxed region is enlarged to the right, highlighting vacuolated myocardial regions. UT; untreated, WT; wild-type, TBG; thyroxin-binding globulin,
